# Giant Primary Schwannoma of the Left Nasal Cavity and Ethmoid Sinus

**DOI:** 10.1155/2016/1706915

**Published:** 2016-06-09

**Authors:** Eugene Wong, Justin Kong, Lawrence Oh, Daniel Cox, Martin Forer

**Affiliations:** ^1^Royal North Shore Hospital, Reserve Road, St Leonards, NSW, Australia; ^2^Faculty of Medicine, University of Sydney, Sydney, NSW 2006, Australia

## Abstract

A unilateral tumour in the nasal cavity or paranasal sinuses is commonly caused by polyps, cysts, and mucoceles, as well as invasive tumours such as papillomas and squamous cell carcinomas. Schwannomas, in contrast, are rare lesions in this area (Minhas et al., 2013). We present a case of a 52-year-old female who presented with a 4-year progressive history of mucous hypersecretion, nasal obstruction, pain, and fullness. Imaging of the paranasal sinuses showed complete opacification of the entire left nasal cavity and sinuses by a tumour causing subsequent obstruction of the frontal and maxillary sinuses. The tumour was completely excised endoscopically. Histopathology was consistent with that of a schwannoma.

## 1. Background

Schwannomas are benign tumours originating from peripheral nerve sheaths. Previous reports indicate that 25–50% of schwannomas occur in the head and neck region, but tumours originating from the nasal cavity or paranasal sinuses are rare, with a reported rate of approximately 4% [1, 2]. In 2001, approximately 40 cases of sinonasal schwannomas had been reported [3]. In 2016, we found just over 100 cases reported in the literature [4].

We report the case of a huge nasoethmoidal schwannoma excised endoscopically.

## 2. Case Presentation

A 52-year-old female presented with a 4-year progressive history of left nasal obstruction, pain, and fullness with intermittent epistaxis. Her symptoms began in China where she described an episode of an upper respiratory tract infection with subsequent development of ongoing mucous production. Other features of her history were otherwise unremarkable.

Findings on nasendoscopy showed a huge left nasal polyp completely obstructing the anterior nasal cavity limiting further examination. On nasendoscopy through the right nostril, the tumour could be seen occupying the left nasopharynx but the right nasal cavity was clear. Examination of the eye and the oral cavity was unremarkable.

## 3. Investigations

**Figure 1:**
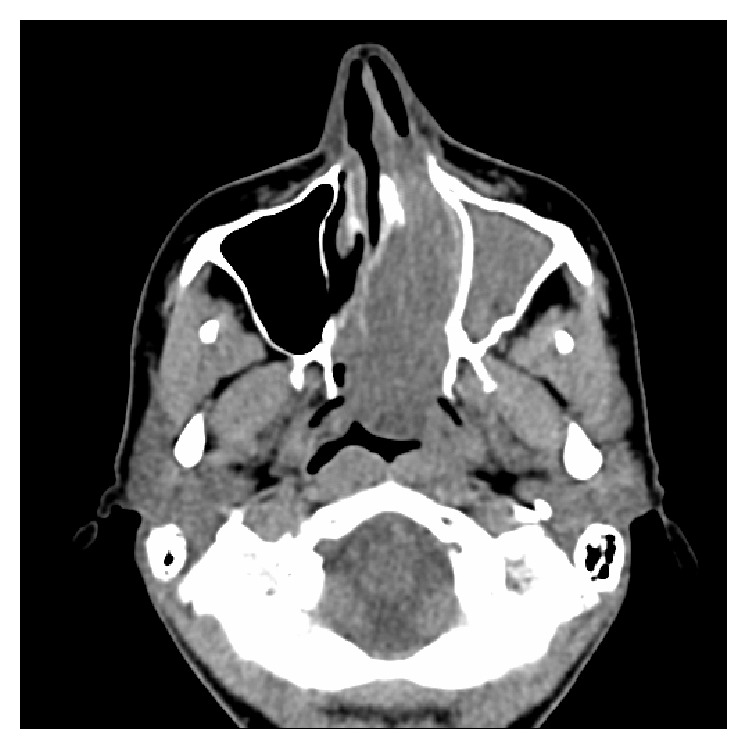
CT (axial view) showing complete opacification of left frontal, ethmoid, maxillary, and sphenoid sinuses and nasal cavity.

**Figure 2 fig2:**
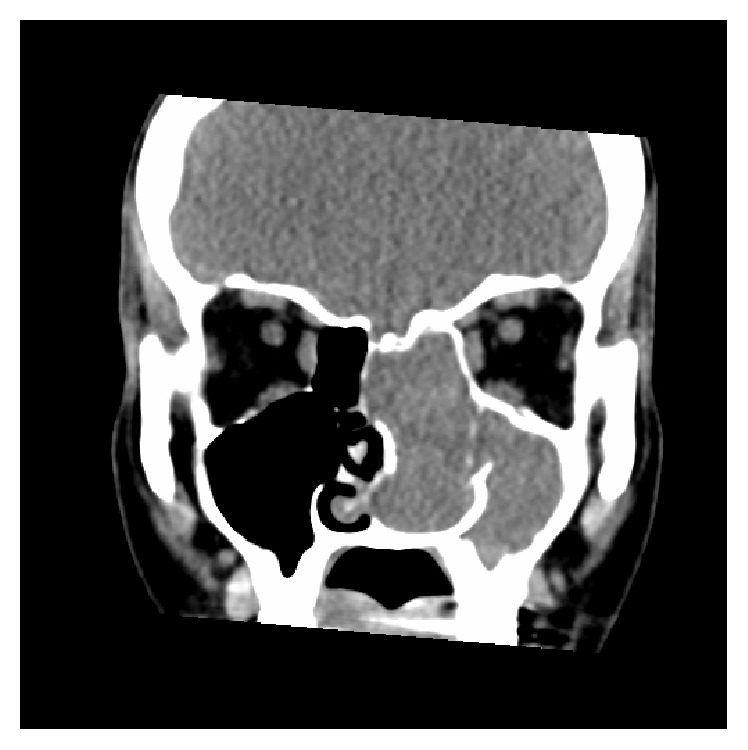
CT (coronal view).

**Figure 3:**
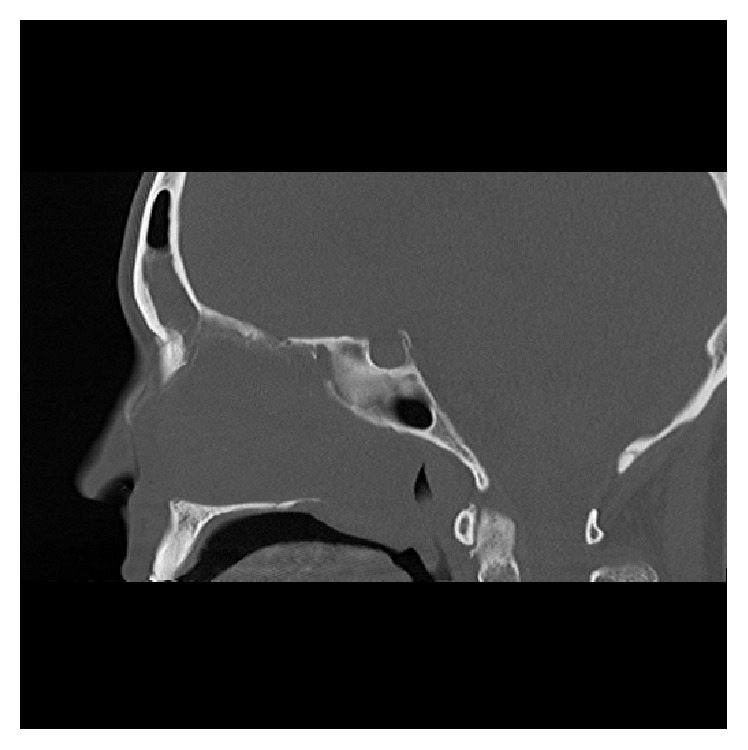
CT (sagittal view bone window).

High resolution CT of the paranasal sinuses showed complete opacification of the left frontal, ethmoidal, maxillary, and sphenoidal sinuses and nasal cavity. The tumour significantly displaced the left lateral nasal wall into the maxillary sinus. There was also hyperostosis of the sphenoid and maxillary sinus walls. The right nasal cavity and sinuses were clear. Axial, coronal, and sagittal views are demonstrated in Figures 1 [Fig fig2]–3.

**Figure 4:**
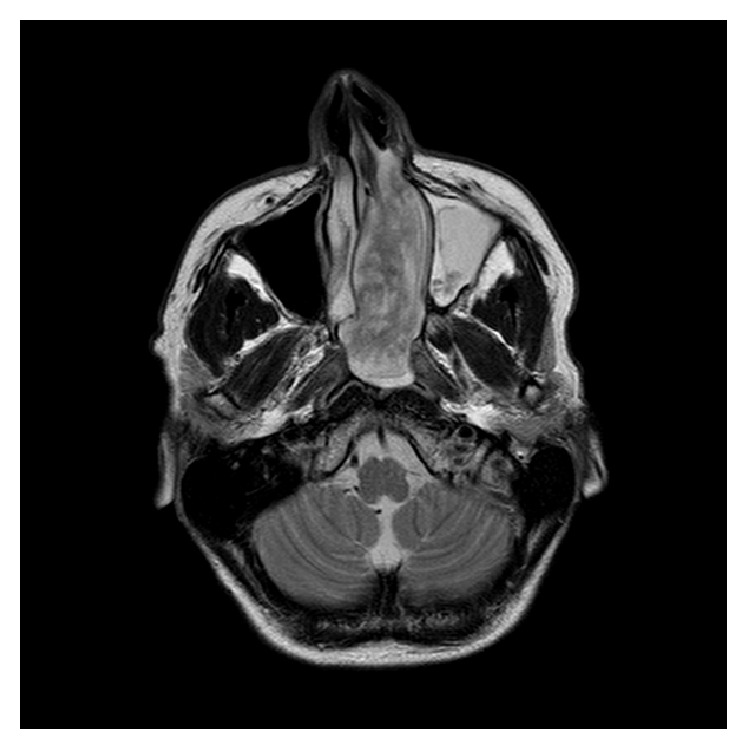
MRI (axial, T2 weighted) showing tumour involving the postnasal space, nasopharynx, and ethmoid air cells and secretions filling the left maxillary antrum and frontal sinus.

**Figure 5:**
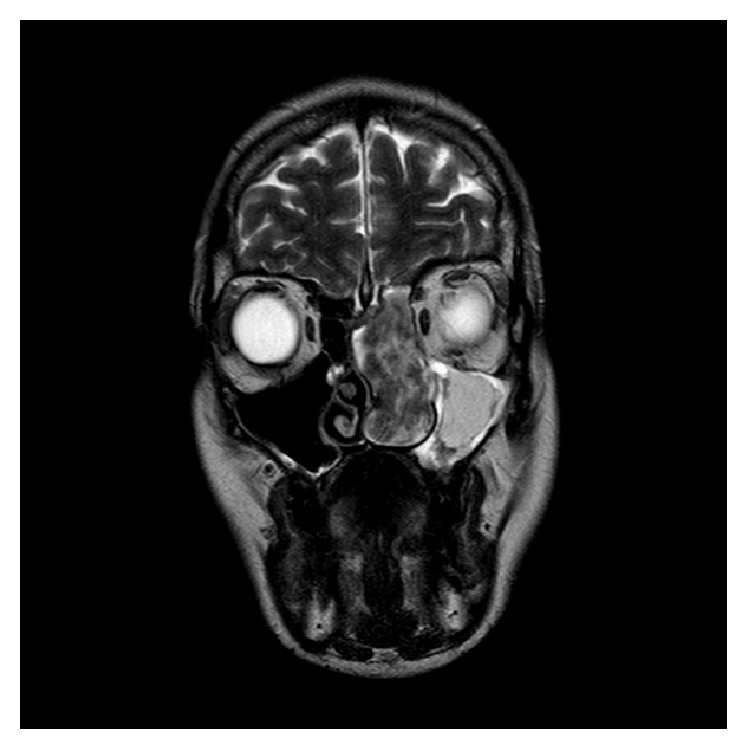
MRI (coronal, T2 weighted).

An MRI was performed to better visualise the soft tissue mass. The mass was identified to fill the entire left nasal cavity, extending into the choana to completely fill the nasopharynx. This mass was measured at 78 × 28 × 51 mm (AP × ML × SI). The mass also occupied the left anterior and posterior ethmoid air cells, obliterating all bony septations. The left maxillary antrum and frontal sinus were filled with secretions secondary to the obstruction caused by the mass. Axial and coronal T2 weighted images are demonstrated in Figures 4-5. Unfortunately post-Gadolinium imaging could not be performed due to patient claustrophobia.

We did not acquire a preoperative tissue biopsy given how promptly a complete excisional procedure could be performed. Furthermore, given the patient's severity of symptoms, complete excision was necessary regardless of the diagnosis, and therefore we felt a biopsy would not have changed immediate management.

## 4. Treatment

Endoscopic resection of the schwannoma was undertaken. The patient was placed under general anaesthetic in a reverse Trendelenburg position. The superior extent of the tumour was dissected off the anterior skull base with no attachment found. Clearance of the frontoethmoidal recess was followed by free evacuation of mucus from the frontal sinus. Inferiorly the tumour was not attached to the nasal floor. Obstruction of the left maxillary sinus ostium was addressed with clearance of the maxillary sinus contents. The tumour was attached posteriorly to the basisphenoid, the point of presumed focal origin. The nasopharyngeal component was easily removed with no attachment found. The floor of the sphenoid sinus was drilled further as the pterygopalatine ganglion was felt to be the likely point of origin and hemostasis was achieved. The tumour was removed in two sections.

The patient was extubated and had an uneventful postoperative recovery.

The two separate sections of the nasal mass were sent for histopathological examination (29 × 25 × 22 mm, 40 × 22 × 20 mm.) Both sections were composed of Antoni-A and Antoni-B areas of variable cellularity consistent with those of a schwannoma. As expected, immunoperoxidase stains for S100 and SOX10 were positive. Tumour was noted to compress thin strips of bone in a submucosal distribution in all margins. There was no evidence of malignancy. Fungal microscopy and culture showed no elements or growth.

## 5. Outcome and Follow-Up

The patient had a very good response to surgery at two-month follow-up, with complete resolution of symptoms. On repeat nasendoscopy, there was no evidence of residual or recurrent disease.

## 6. Discussion

Unilateral tumours in the nasal cavity causing nasal obstruction, pain, fullness, and epistaxis are usually caused by benign disease processes such as polyps, cysts, and mucoceles. A unilateral tumour originating from the nasal cavity should also stimulate the consideration of the rare esthesioneuroblastoma, a neoplasm originating from the olfactory neuroepithelium that has significant heterogeneity in management and variation in prognosis [5].

Schwannomas of the nasal cavity and sinuses produce similar symptoms but are much rarer. Previous reports suggest that sinonasal schwannomas represent less than 4% of all head and neck schwannomas, with only approximately 40 cases reported as of 2001 and 100 cases as of 2014.

Schwannomas are benign tumours of peripheral nerve sheaths, and it has been proposed that sinonasal schwannomas may originate from the ophthalmic or maxillary branches of the trigeminal nerve or from sympathetic or parasympathetic fibres from the carotid plexus or sphenopalatine ganglion [6]. The majority of patients present with progressive nasal obstruction with pain, headache, and epistaxis but can occasionally cause ptosis, proptosis, or diplopia.

The diagnostic workup for sinonasal schwannoma should include nasendoscopy, CT, and MR imaging of the paranasal sinuses to examine the extent of disease and to guide surgical approach for excision. As most schwannomas have focal origin and are in the most part encapsulated, these tumours are commonly amenable to endoscopic resection.

This case report highlights the need for schwannoma to be included in any differential diagnosis of any soft tissue mass of the sinonasal spaces.
